# Integrated UWB/MIMU Sensor System for Position Estimation towards an Accurate Analysis of Human Movement: A Technical Review

**DOI:** 10.3390/s23167277

**Published:** 2023-08-19

**Authors:** Vinish Yogesh, Jaap H. Buurke, Peter H. Veltink, Chris T. M. Baten

**Affiliations:** 1 Roessingh Research and Development, Roessinghsbleekweg 33B, 7522 AH Enschede, The Netherlands; j.buurke@rrd.nl (J.H.B.); c.baten@rrd.nl (C.T.M.B.); 2 Department of Biomedical Signals and System, University of Twente, Drienerlolaan 5, 7522 NB Enschede, The Netherlands; p.h.veltink@utwente.nl

**Keywords:** integrated UWB/MIMU, data fusion, position estimation, human movement analysis, position accuracy

## Abstract

Integrated Ultra-wideband (UWB) and Magnetic Inertial Measurement Unit (MIMU) sensor systems have been gaining popularity for pedestrian tracking and indoor localization applications, mainly due to their complementary error characteristics that can be exploited to achieve higher accuracies via a data fusion approach. These integrated sensor systems have the potential for improving the ambulatory 3D analysis of human movement (estimating 3D kinematics of body segments and joints) over systems using only on-body MIMUs. For this, high accuracy is required in the estimation of the relative positions of all on-body integrated UWB/MIMU sensor modules. So far, these integrated UWB/MIMU sensors have not been reported to have been applied for full-body ambulatory 3D analysis of human movement. Also, no review articles have been found that have analyzed and summarized the methods integrating UWB and MIMU sensors for on-body applications. Therefore, a comprehensive analysis of this technology is essential to identify its potential for application in 3D analysis of human movement. This article thus aims to provide such a comprehensive analysis through a structured technical review of the methods integrating UWB and MIMU sensors for accurate position estimation in the context of the application for 3D analysis of human movement. The methods used for integration are all summarized along with the accuracies that are reported in the reviewed articles. In addition, the gaps that are required to be addressed for making this system applicable for the 3D analysis of human movement are discussed.

## 1. Introduction

Currently, ambulatory 3D analysis of human movement finds an ever-expanding range of applications in rehabilitation medicine, physical therapy, sports, and ergonomics. The most widely used wearable sensor modality is the Magnetic Inertial Measurement Unit (MIMU) which has gained popularity due to its low cost, ease of use, and portability [1,2]. MIMUs typically comprise a 3-axial linear accelerometer, rate gyroscope, and magnetometer that can simultaneously measure 3D linear acceleration, angular velocity, and the local magnetic field vector in the sensor reference system, respectively [3]. MIMU sensor systems are typically capable of accurately estimating 3D orientation and, by extension, all other 3D rotational kinematics, in a world reference frame by exploiting the redundancy in angular movement data from these three sensor modalities through data fusion algorithms such as the Extended Kalman Filter (EKF) [2,4,5].

However, the accuracy is limited when the observability of the MIMU sensor’s two natural references, which are exploited to relate the sensor reference system to the world reference system, is sub-optimal. These two natural references are the earth’s gravity and magnetic fields. Observability of the earth’s gravity is only threatened in situations of (prolonged) large or varying free acceleration, like situations of humans in moving vehicles (especially in curves) and in some performance sports (e.g., speed skating). In applications of physical therapy and rehabilitation, this typically does not occur. Observability issues of the earth’s magnetic field typically occur in the vicinity of ferromagnetic materials and easily occur in all applications, especially indoors and with sensors close to any constructed floor [6,7]. Also, 3D linear displacement kinematics relative to a starting position can be derived. However, the mostly consistent accuracy achieved for 3D angular kinematics cannot be achieved in 3D position estimation, as this involves double integration of the acceleration signal, causing strong integration drift [8,9]. Since neither rate gyroscope nor magnetometer provide additional displacement data, there is no possibility to counteract this drift through data fusion methods, as is performed in the angular estimates. As a consequence, the displacement estimates and the derived position estimates are susceptible to relatively large errors.

Multiple attempts to reduce these errors were made by exploiting assumptions on the movement performed. This is realized, for example, by assuming an instant of zero velocity of the foot in the stance phase of walking in a method called the Zero-Velocity-Update (ZUPT) approach [10,11] or by assuming constraints to the joint 3D kinematics based on a biomechanical model of (part of) the human subject [12]. The application of these methods is (severely) limited by the validity of their assumptions regarding the individual subject’s biomechanics or behavior or situational conditions. As the accuracy problems are due to integration drift, other methods are proposed to limit positional estimates to estimates relative to temporary world frames in cyclical movements short enough to prevent integration errors from becoming substantial [13]. Exploiting the above assumptions can be a solution in some applications, but, especially in patients, they have limited applicability as patients typically perform in an individual-specific pattern which severely violates these assumptions.

Another possible solution suggested is the addition of additional sensor modalities to the MIMU, seeking to create redundancy for 3D positional data. This would again enable a data fusion approach to tackle the integration drift issues and does not require any assumptions on the subject’s biomechanics or behavior, or the situation and/or the short-term cyclicity of the activity, specifically facilitating accurate 3D analysis of human movement in patients. This approach requires adding synchronized ranging sensors to the MIMUs that record the absolute distance between the MIMUs on-body or between MIMUs on-body and reference ranging sensors positioned around the subject. For a successful application in the 3D analysis of human movement, the position estimates by data fusion of MIMU with some additional sensor modality would require an accuracy that is similar to vision-based systems, which are currently regarded as the ‘gold standard’. The accuracy of the vision-based systems is considered to be clinically relevant and sufficient enough for clinical decision-making [14,15]. This then implies that the position estimation errors of data fusion should be of sub-centimeter level (ca. 1 cm error or better) as it is for the vision-based systems [16,17]. In addition, a sufficiently high update rate faster than 1 Hz from the position sensor being used for data fusion is necessary to keep the integration drift errors low [18].

Commonly proposed ranging sensor methods for the estimation of positions in indoor space exploit ultrasound [19] or infrared (IR) sensors [20]. However, ultrasound is not suited for continuous 3D analysis of human movement due to the low-frequency band [21,22], while the IR-based solutions require a direct Line of Sight (LOS) which severely limits their use for wearable 3D analysis of human movement [19,21]. Alternate technologies suggested for ranging are Wi-Fi, ZigBee, Bluetooth, Radio Frequency Identification (RFID), and Ultra-wideband (UWB) [23,24,25,26,27,28]. Among these UWB is developed with the explicit purpose of ranging, while all the others were developed for communication purposes and only later were they used in ranging applications. Also, of all these methods the highest accuracies are reported for UWB ranging solutions (errors < 10 cm) [29,30,31,32], with lower accuracies reported for the other solutions [33,34]. These UWB sensors are developed as miniature ranging devices with smart sensor clock-independent Two-Way-Ranging (TWR) algorithms. They use extremely low power and are currently available in single-chip-based packaging [29]. However, their accuracy is still limited, especially in Non-Line-of-Sight (NLOS) conditions [31].

Both MIMUs and UWB have their limitations in accuracy when independently used for the estimation of the (relative) 3D position. However, as the sources of their errors are complementary, it is expected that a data fusion-based integrated estimator would improve position estimation accuracy to a higher level than possible with either of them separately. This is because the MIMUs are prone to drift, but not affected by NLOS, while UWB provides an absolute estimate of distances that is drift-free, but is affected by NLOS. In the last decade, many researchers have exploited the complementary characteristics of UWB and MIMU to achieve accurate position estimates by smart data fusion [35]. All this suggests that, if an accuracy similar to the current lab-based ‘gold standard’ can be achieved with the combination of UWB and MIMU in a set of on-body sensors, applying a data fusion-based approach could have great potential in improving ambulatory 3D analysis of human movement. However, to the knowledge of the authors so far, UWB/MIMU data fusion applications involving humans are only reported in pedestrian tracking and localization applications. No reports on exploitation for the full-body 3D analysis of human movement with on-body integrated UWB/MIMUs were found by the authors.

To be able to research and develop such an application for 3D analysis of human movement, there is a need to identify the current state-of-the-art research on integrated UWB/MIMU methods for position estimation. Also, a better understanding of the strengths and weaknesses of UWB/MIMU-based estimation along with the opportunities and threats for successful application in the 3D analysis of human movement with only on-body UWB/MIMUs is essential. To the knowledge of the authors, no comprehensive literature review currently exists that provides the current state-of-the-art research on position estimation of humans (on-body) using these integrated UWB/MIMU sensor systems. Therefore, this paper is intending to answer these questions through a technical survey of the literature and provide a summary of methods and algorithms reported in the peer-reviewed literature so far, along with the accuracies and stabilities achieved.

Thus, the main aim of this paper is to identify the possibilities and limitations of integrated UWB/MIMU sensor systems for achieving accurate position estimates in 3D analysis of human movement applications. This is achieved through a comprehensive technical review of the literature in the past decade. To address this aim, several objectives were formulated:

1. To provide an overview of the current state-of-the-art research on estimation methods of (relative) 3D/2D positions of the human body, human body segments, or joints applying data fusion methods integrating UWB and MIMU sensor technology.
2. To summarize the configurations, protocols, and algorithms used in these estimation methods.
3. To summarize the achieved accuracy and stability reported for these methods.
4. To discuss the strengths and limitations of these methods and their consequences in the context of future application of 3D analysis of human movement.

## 2. Methods

### 2.1. Search Procedure

The literature search was conducted in the databases of Scopus (Elsevier), Web of Science and PubMed on 2 February 2023. To frame the appropriate search terms, a pre-search was carried out where a list of keywords was identified by considering a set of the most relevant articles that were to be included in the review. Search terms were then grouped into four groups, where the groups were combined using the AND operator, while within the group the terms were combined with the OR operator. The search terms are listed below:

- Group 1: UWB, ultra-wideband;
- Group 2: IMU, inertial measurement unit, IMMU, inertial magnetic measurement unit, MIMU, magnetic inertial measurement unit, inertial sensors, INS, inertial navigation system, inertial motion capture, accelerometer, gyroscope;
- Group 3: indoor positioning, position, indoor localization, localization, tracking, navigation, indoor navigation, trajectory tracking, distance, motion analysis, movement analysis, dynamic activity, ambulatory, posture, pose, orientation;
- Group 4: sensor fusion, data fusion, filtering algorithm, fusion, filter, Kalman filter, graph optimization, particle filter.

### 2.2. Study Selection

Followed by the initial search of the literature, the title and abstracts of all the articles found were screened for the inclusion and exclusion criteria. The articles were included when they satisfied the following inclusion criteria:

1. The journal article was published within the last decade, that is between the years 2012 to 2023.
2. The research used the integrated UWB and MIMU sensors alone for estimating positions.
3. The research included experimental validation of the position estimation methods, which were validated against a standard reference system (vision-based systems such as VICON or other means where the ground truth is known).
4. The sensor system was applied for human movement tracking, analysis of human movement, or human localization.
5. The data fusion was based on UWB ranging or position estimates that used multiple sensor nodes instead of localization based on a single UWB sensor that uses the reflected signals similar to radar.

The articles are excluded if:

1. They were not written in English.
2. Additional sensors were used in data fusion along with the UWB/MIMU combination.
3. They were conference proceedings, abstracts, review articles, or letters to editorial.
4. They were applied or used for non-human situations such as drones, robots, etc.

The first two exclusion criteria were straightforward and were to align with this review’s goal. The third exclusion criterion for excluding conference proceedings was due to a significant overlap in the data fusion approaches with the journal articles. In addition, these conference articles had limited experimental validation. Therefore, this choice of exclusion ensures clarity and quality of this review. For the fourth exclusion criterion, the focus is exclusively on human body situations, which is to align with this review’s objective of assessing the applicability of the existing literature for accurate 3D analysis of human movement. Also, there is a unique effect on ranging accuracy due to on-body placement [33] and thus this criterion for exclusion also ensures a targeted exploration of methodologies relevant to the intended use-case scenario.

Full-text screening of all the shortlisted articles was performed to validate the inclusion and exclusion criteria and was then reviewed by the researcher (VY).

### 2.3. Data Analysis

The included articles were analyzed in detail to extract information on the methods and their accuracies and stabilities. The information extracted is summarized in the Section 3. Regarding the achieved accuracy in ranging, the main parameters extracted were Mean Absolute Error (MAE) and Root Means Square Error (RMSE) along with minimum and maximum errors, if available. Reported Mean Square Error (MSE) values were converted to RMSE for consistent reporting in the tables. Also, if the RMSE and MAE values were reported for the individual axes, then the vector norm of the errors in each axis is computed and also depicted in the table for better comparison to other articles. For articles with the error values not explicitly indicated, it was extracted from the error plots. To facilitate comparison of an accuracy reported through MAE with one reported through RMSE the reader may use the following relationship: RMSE is equal to $\sqrt{\pi /2}$ times MAE, valid when the errors may be assumed to be uniformly distributed [36], where $\sqrt{\pi /2}\approx 1.5$.

## 3. Results

### 3.1. Search Results

**Figure 1:**
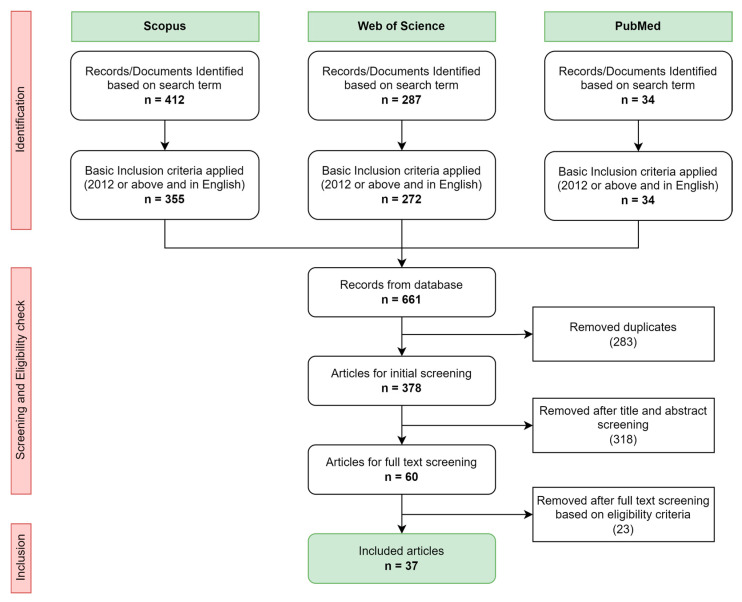
Review process flow diagram and inclusion results.

The literature search resulted in the identification of 661 articles in total from all three databases. The flow chart in Figure 1 highlights the results of each stage of the reviewing process. The identification of duplicates, initial title/abstract screening and selection of articles were performed using the Rayyan web application (Rayyan System Inc, Cambridge, MA, USA), an intelligent collaborative research tool for literature reviews. The final included articles were then exported to the reference manager software EndNote X8.2.

**Figure 2:**
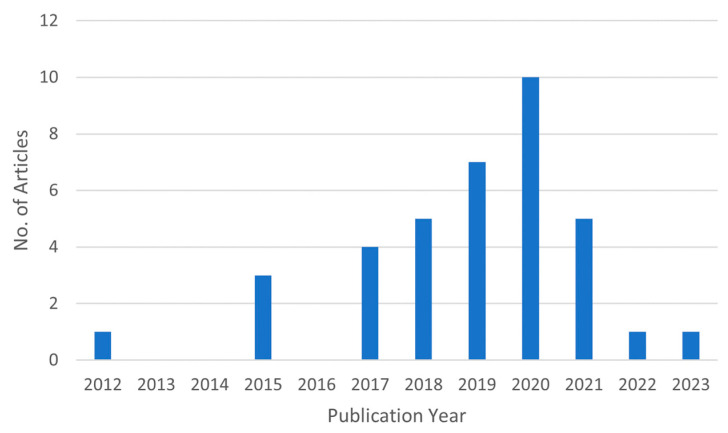
Number of relevant publications found per year.

Out of the total 661 articles, the duplicates (283 records) were removed resulting in 378 records for the initial screening stage. The initial screening stage was based on the content of the title and abstract, which resulted in eliminating 318 articles based on the inclusion/exclusion criteria. The articles excluded were based on varying reasons which include: being non-journal articles (209 articles), being not applied/used for human movement (47 articles), using additional sensors along with the UWB and MIMU used for the data fusion (28 articles), not using either UWB or MIMU in data fusion (24 articles), being reviews/background paper (5 articles), applying only single UWB sensor ranging based on reflected signals (3 articles), not used for position estimation or localization (1 article), and written in another language (1 article). The full text was extracted for the remaining 60 articles to perform full screening and assessment of eligibility. With full-text screening, 23 articles were excluded due to lack of experimental evaluation (4 articles), the full-text being unavailable (3 articles), UWB not being used for data fusion (1 article), and for not being tested/validated on human subjects or other applications (14 records). Finally, a total of 37 articles were included for analysis in this review. A plot of the number of articles published over the years is provided in Figure 2. For the year 2023, the number of articles is only based on the first 5 weeks of the year.

### 3.2. Literature Overview of Integrated UWB/MIMU Sensor System

A summary of all the major data that were extracted from the included records for review is provided in Table 1. The extracted information includes the year of publication, sensor specifications, the configuration of the sensors, placement of the sensors on the body, the algorithm used for sensor fusion (method/approach), update parameter used for the fusion, and information regarding sensor hardware integration (separate units, physically attached, or integrated hardware platform). Information that is unavailable or not specified by the articles is marked as “-” in the table.

In the following subsections, the data fusion of the UWB and MIMU reported in the reviewed articles are summarized. The data fusion of the two sensor systems in the articles reviewed consists of a prediction phase where information from one of the sensors is used to estimate the position, which is then followed by an update phase where the information from the additional sensor/s is used to correct for the errors in the estimation of position in the prediction phase. Therefore, the position estimation methods in each of the sensor systems are first described along with their configurations (Section 3.2.1 and Section 3.2.2 for UWB and MIMU, respectively), and sensor placement strategies (Section 3.2.3) are described individually. Followed by this, the combination strategy used by the articles for the sensor data fusion is described (Section 3.2.4). If additional improvements in the algorithm for NLOS dealing were reported in the reviewed articles, they are also described (Section 3.2.5).

#### 3.2.1. UWB Sensor System Configuration and Measurement

The most commonly used UWB sensor hardware system is the DW/M1000 OR DMW1001 (DecaWave, Dublin, Ireland) [31,32,33,35,44,46,47,50,51,57,58,59,60,61,62,65,66,67], which was used in 18 articles, while the Unisense series 7000 was used in 4 articles [7,38,39,41]. The PlusOn 410 UWB [34], UWB Mini4sPlus [54], and BeSpoon [43] were each used in one article. Twelve articles did not report on the UWB sensor that was used in their study [37,40,42,45,48,49,52,53,55,56,63,64]. Detailed information on the sensor systems used, along with their update rates, is provided in Table 1. UWB sensors are henceforth referred to as ‘nodes’, and those used in these articles were classified into two classes based on their functionality and named accordingly as ‘anchors’ and ‘tags’. UWB nodes are referred to as anchors when they are placed in fixed known positions around the measurement region and typically form a frame of reference for the position of the tags, while tags are the UWB nodes with an unknown, dynamically changing, position that is worn by the subject [65].

**Figure 3:**
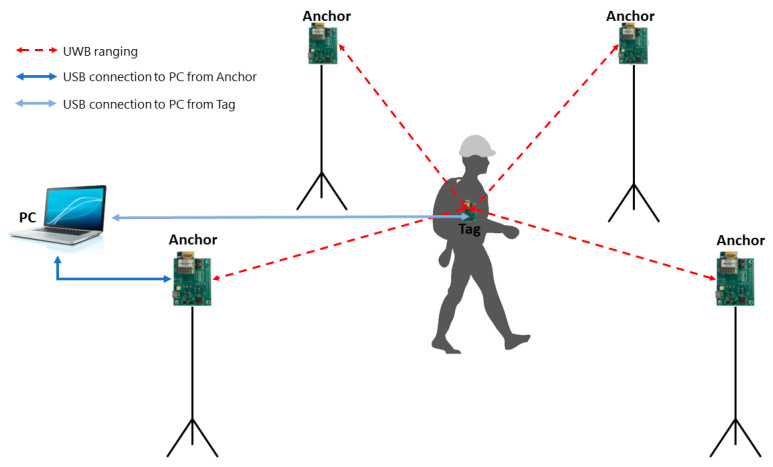
Star UWB sensor system configuration.

All the articles reviewed use a ‘star topology’ for the ranging operations (Figure 3). In this topology for each tag, the ranging operations only take place between this individual tag and each anchor, while no ranging operations are performed between tags or between anchors. The alternative ‘swarm topology’ (a topology in which ranging is performed between all node pairs) is not used in any of the articles.

The distances between all of the wearable tags and the fixed anchors were estimated using a variation of the TWR scheme that utilizes the Time of Arrival (ToA) information. The typical TWR scheme is described in detail in [68]. The resulting distance estimates between the tags and anchors were then used to estimate the 2D or 3D position of the tag by the method of trilateration or multilateration, or by an optimization method minimizing least square position errors [69]. For both methods, at least the (required) minimum of three anchors was used to locate each tag in 2D space and at least the (required) minimum of four anchors was used to locate a tag in 3D space [65]. However, in some of the reviewed articles the UWB distance estimates were used as a direct input for their data fusion approach. For these approaches, less than three anchors was enough as there is no need for trilateration to compute positions.

Among the reviewed articles, the number of fixed anchors ranged from 1–10, where the majority of the articles (32 articles) used 3–5 anchors. Two of the articles used seven anchors [53] and ten anchors [40], respectively. Both the articles that used more than five anchors, stated that an increase in the number of anchors creates redundancy in the number of ranging, thereby increasing the robustness against situations of NLOS between one or more node pairs. In four articles, the number of anchors was limited to two anchors [57,58] or one anchor [35,47]. These four articles with fewer than three anchors, used only the distance estimates for data fusion and did not estimate positions from UWB. The number of tags on the subject for tracking the motion was always one, except for Zihajehzadeh et al., 2017 [7], where three tags were used. In this article, the additional two UWB tags were used for computing the facing direction (heading) of the subject’s body in the horizontal plane while only the third one was used for position estimation.

#### 3.2.2. MIMU Sensor System Configuration and Measurement

A wide range of MIMUs was used in the reviewed articles and the most commonly used MIMU sensor hardwares are Xsens IMU (Xsens BV, Enschede, Netherlands) [7,38,39,40,41], iPhone inbuilt IMU [47,50,57,58,67], and MPU9250/9150 (InvenSense Inc, San Jose, CA, USA) [31,33,44,53,63], which were each used in five articles. Custom-made MIMUs with individual sensors (accelerometer: ADXL203, gyroscope: ADXRS620, and magnetometer: HMC5983) [46,49,60,64] were used in four articles. Other MIMUs used were MPU6050 (InvenSense Inc, San Jose, CA, USA) [61,65], EBIMU-9DOF [62], JY901B [52], and Starneto [34]. Also, two articles had a custom-built IMU with sensors ICM20602 (Invensense, USA)/IST8310 (iSentek Inc, Taipei, Taiwan) [59] and LSM330DLC (STMicroelectronics, Geneva, Switzerland)/HMC58832 [51]. Two articles used an IMU without a magnetometer, namely LIS3DH (STMicroelectronics, Geneva, Switzerland) [35] and JY61 attitude sensor [54]. All of the MIMUs communicate wirelessly or via a USB connection to a PC or a recorder carried by the subject. The majority of the included articles used a single MIMU on the subject except for four articles that used more than one MIMU [7,41,42]. Eight articles did not report on the MIMU sensor that was used in their study [32,37,42,45,48,55,56,66].

**Figure 4:**
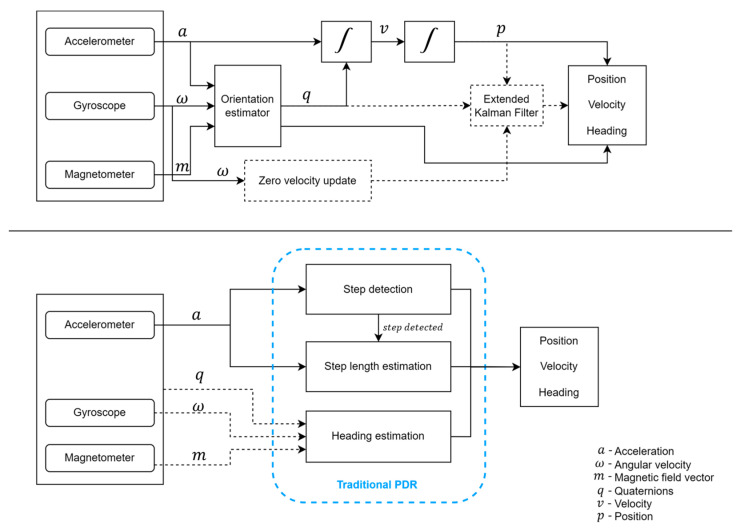
Schematic of the MIMU position estimation algorithms applied; Integration method (**top**) and traditional Pedestrian Dead Reckoning (PDR) algorithm (**bottom**).

Two types of position estimation methods were utilized for the MIMU sensor systems in the reviewed articles. The first method, namely the ‘integration method’, estimates the MIMU node displacement relative to the start position by double integration of its free acceleration signal transferred to a global inertial reference frame. This transformation needs the estimated orientation of the MIMU node. Articles in this review using this method did estimate these orientations by data fusion of sensor acceleration, angular velocity, and magnetic field vector data or by data fusion of only sensor acceleration and angular velocity. In addition, some of the articles use the ZUPT algorithm and EKF for improving the position estimates for the IMU placed on the foot, exploiting typical properties of the cyclical movement of the feet in walking. This is illustrated in Figure 4, where the blocks with solid lines apply for all while the dotted lines are applicable for the ones that were relying on the additional ZUPT algorithm and EKF.

The second method used was the Pedestrian Dead Reckoning (PDR) method, where the algorithm detects the heel strike instants and then computes the amount of displacement of the sensor node during each step (heel strike to heel strike) and the direction of displacement separately. The position at the end of the step was then estimated by adding the estimated displacement to the position estimate at the beginning of the step in the estimated direction [42]. A schematic diagram of the PDR algorithm is provided in Figure 4. It illustrates that the heel strike instant and the step length were computed based on the acceleration of the sensor, while the heading angle was estimated from either the MIMU orientation, the magnetometer, or through data fusion of all the combined information (as illustrated with dotted lines in Figure 4). The reviewed articles that relied on the PDR algorithm for estimating the position [42,43,47,51,52,57,58,59,61,62,67] using MIMUs had all adapted the same basic algorithm or with minor improvements. Researchers who are interested to know more details on the general PDR algorithm are referred to [42].

#### 3.2.3. Placement Location of the Sensors on the Body

In the reviewed articles, the UWB and MIMU sensors on the body were not always integrated into a single sensor platform or placed physically tied to each other. The UWB and MIMU were placed at different locations on the body for 15 articles [31,32,37,39,42,44,45,46,48,49,53,55,56,60,64]. While for another 19 they were physically tied to each other and thus placed at the same location [7,33,34,35,38,40,41,43,47,50,54,57,58,59,61,62,63,65,67]. Among these nineteen articles, only four articles [33,35,59,61] had the MIMU and UWB sensor hardware integrated into a single sensor platform with one central onboard microcontroller. The placement data were not available for three articles [35,51,66].

**Figure 5:**
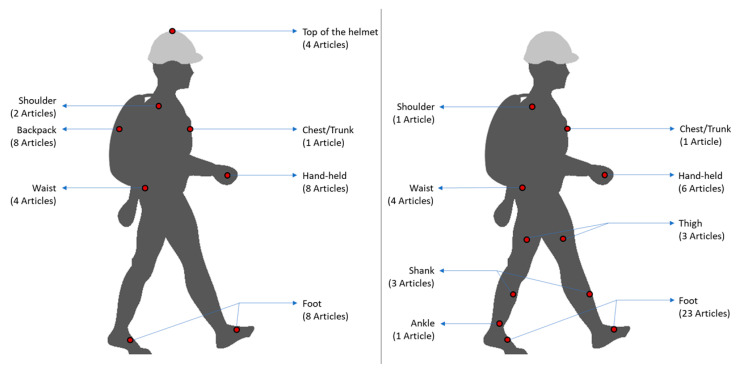
UWB sensor placement (**left**) and MIMU sensor placement (**right**) locations on the human subject in the reviewed articles along with the number of corresponding articles for each location.

As previously mentioned in Section 3.2.1, only one UWB tag was placed on the body and was placed mainly on the foot [33,34,40,54,61,63,65], on a shoulder backpack [45,46,48,49,55,56,60,64], or held in hand steady with no swinging [37,43,47,50,52,57,58,67]. Meanwhile, other articles had a placement on the waist [38,39,41], on a head-worn helmet [31,32,44,53], on the shoulder [42,59], or on the chest/trunk [62]. Zihajehzadeh et al. 2017, [7] had three UWB sensors that were placed on the waist and both feet. The mounting locations of the UWB sensors on the body are illustrated in Figure 5 (left) with the number of corresponding records.

For MIMU sensors, the most widely used placement location was the foot [31,32,33,34,40,42,44,45,46,48,49,53,54,55,56,60,61,63,64,65] and it comprised 20 articles. Among them, one article [42] had two sensors on both feet while others were a single MIMU on one of the feet. The other placement locations were ankles [37], waist [38], shoulders [59], chest/trunk [62], or held in hand [43,47,50,57,58,67]. Three articles [7,39,41] had a set of seven MIMUs placed such that there was one on the waist, and pairs of two on the upper legs, lower legs, and feet. These additional MIMUs were used to estimate the body kinematics such as angles and pose in addition to the general body position estimation. For four articles no placement information was available [35,51,52,66]. The placement locations of MIMUs on the body for all the articles are illustrated in Figure 5 (right).

#### 3.2.4. Position Estimation Methods Combining UWB/MIMU Data

The key objective of the data fusion approach in examined papers was to achieve a better position estimate than what can be achieved with only MIMU-based methods or only UWB-based methods by combining the strengths of both and, with that, overcoming their weaknesses. The data fusion approaches used in the reviewed articles can be widely classified as loosely coupled and tightly coupled approaches, based on the way the data were used for the UWB/MIMU fusion. The loosely coupled approach uses the raw time of UWB transmissions between the nodes (distance estimates), while the tightly coupled approach uses the triangulated position estimates of the UWB for the data fusion. All the algorithms/methods identified are listed in Table 1, along with the update parameter which directly indicates if it is a loosely or tightly coupled approach.

Summarizing the methods in the articles reviewed, the general data fusion pipeline generally contained two stages, which were a data preprocessing and a data fusion stage. Data preprocessing stages included activities like setting the start position, a priori estimation of the error characteristics of the sensor output, and detection of zero velocity instants. The data fusion stage had two phases. In the first phase (prediction) the position of the sensor (in loosely coupled methods) or distances between sensors (in tightly coupled methods) were estimated based on information from one of the two sensors used in the experiment using the algorithms described in Section 3.2.1 or Section 3.2.2. In the second phase (update) the additional redundant information, here the second type of data, was merged with the predicted/priori estimates to achieve a more accurate estimate.

All the articles reviewed except [50] used MIMU sensor information in the prediction phase and UWB in the update phase, probably due to the typically higher sampling rate of the MIMUs and since quantifying error characteristics was easier for UWB data. The one paper that used UWB data in the prediction phase [50], used MIMU in the update phase only to get the relative orientation of the two ranging UWB. This was subsequently used to correct the UWB ranging error previously characterized in this article based on the orientation of the ranging operation. As mentioned in Section 3.2.2, all the listed articles with the integration method used that approach during their prediction phase, while the articles listed under PDR utilized the PDR algorithm for estimating positions in the prediction phase. In the update phase, articles listed under the loosely coupled approach used positions as the UWB observation, while the ones under the tightly coupled approach used distances as the UWB observation. The estimation algorithms of the UWB were as mentioned in Section 3.2.1.

**Figure 6:**
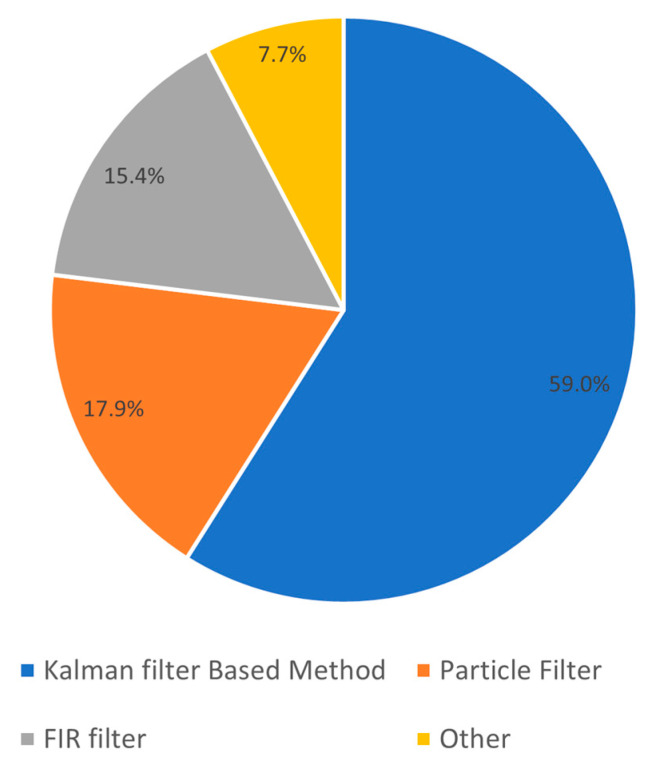
Distribution of data fusion methods for position estimation.

The reviewed papers most commonly used Kalman Filter (KF)- or Particle Filter (PF)-based methods for data fusion (Figure 6). Almost 59% of the articles reviewed utilized the KF-based data fusion methods [7,33,34,35,37,38,39,41,42,43,48,51,52,53,54,59,61,62,63,64,65,66]. Among the Kalman Filter-based articles, thirteen articles used a loosely coupled approach [7,33,34,37,38,39,41,51,52,54,62,65,66], while nine articles used the tightly coupled approach [35,42,43,48,53,59,61,63,64]. All articles using KF follow the general data fusion pipeline as mentioned above, which optimally combines the position estimates from MIMU and UWB by calculating a weighted average of the predicted state and the updated measurement considering their uncertainties. The main variations seen among them are based on the use of multiple layers of KF namely the cascaded KF [7,38,39,41], or a different tuning approach of the KF covariance or error parameters. The multiple layered or cascaded KF consisted of independent KFs, where each KF was performing a data fusion for estimating orientation, position, and heading, which were performed in order. Additionally, some articles use an EKF [35,37,43,51,52,53,54,61,63,65] or an Unscented Kalman Filter (UKF) [35,42] for dealing with non-linear models.

Approximately 18% of the articles relied on the particle-filtering approach for the fusion of the two systems [31,32,47,50,57,58,67]. Articles using the PF method represent the position estimates from MIMU as a set of particles except for [50] which uses UWB distances (as described previously). These particles were propagated using dynamic models based on the UWB update measurements and their weights were updated based on their closeness to observations from UWB. The particles were then converged by resampling the particles with higher weights. The PF method is reported to be better in handling non-linear and non-Gaussian systems in the reviewed articles. Among the PF-based articles, two articles used a loosely coupled approach [31,32] while five articles used a tightly coupled approach [47,50,57,58,67].

The Finite Impulse Response (FIR) filtering-based approach was utilized for approximately 15% of the reviewed articles and they were all from the same author or research group [45,46,49,55,56,60]. FIR filter-based approaches combine the information from UWB and MIMU by convolving their measurements with specific filter coefficients which are based on the sensor measurement characteristics. They all exploit the temporal properties of the FIR filter. The FIR filters in the reviewed articles either used Extended Finite Impulse Response (EFIR) filter [45,55,60] or Unbiased Finite Impulse Response (UFIR) filter [46,49,56] based approaches. Where the EFIR method mitigates the errors by assigning appropriate weights to the measurements from both sensors, the UFIR considers the characteristics of both the sensors and constructs multiple FIR filters that effectively eliminate the errors.

Three other methods were also found, namely graph optimization-based fusion [44], Maximum a Posteriori (MAP) estimation algorithm [40], and a combination filter with KF and PF [31]. The graph optimization approach represents the sensor measurements and their relations as a graph. In the reviewed article using graph optimization [44], the UWB anchors are represented as vertexes of the graph and the information from UWB and MIMU measurements are used to represent the constraints on each vertex. An optimization is performed on this graph minimizing the cost function. Finally, this method determines the confidence level for both the sensor observations based on the optimization results and the combined measurement errors. In the article on the MAP algorithm [40], both the sensors provide a likelihood function that informs how likely the target states are given and is used to model the measurement model. The MAP algorithm finds the state that maximizes the probability of the posterior by performing an optimization that considers the sensor measurement model and prior information. The combined KF and PF filter approach article [31] uses an EKF for estimating position from MIMU as described in Section 3.2.2 and then used these position estimates for a PF approach.

#### 3.2.5. Non-Line of Sight (NLOS) Mitigation Strategies

In the reviewed articles, 14 articles [34,43,50,51,52,53,54,59,61,62,63,65,66,67] had some NLOS mitigation strategies in their algorithm. In all articles, the NLOS mitigation strategy first involved the identification of the NLOS situation, followed by the NLOS error elimination. The NLOS identification methods used can be mainly classified into two types. The first class of detection methods was based on communication channel characteristics [51,59,61,62,66,67]. Here, all of the methods relied on the fact that the Received Signal Strength (RSS) of the multi-path is smaller than the RSS of the direct path. The articles using this principle then used a threshold for this difference between the two RSS to classify the measurement as LOS or NLOS except for [66], which used a state vector machine for classification based on the channel characteristic information.

The second class of methods for detecting NLOS situations was based on the ranging estimation inconsistencies [43,52,53,54,63,65]. Here, the ranging estimations were used to either obtain the Mahalanobis distance for estimating outliers [43,53], the likelihood ratio test [52,54], or residual errors between the ranging estimate and MIMU estimates for each instance to identify outlier or NLOS [63,65]. In addition to the two methods mentioned above, two articles used different approaches, where one of them [34] used distance estimates from the anchors only in front of the subject carrying sensor with LOS. Meanwhile, the other [50] used MIMU to find the orientation of the UWB tag to anchor and used a predefined error model based on the facing orientation between the tag and anchor. The NLOS error elimination in all these identified articles was performed by adjusting the error covariances for the data fusion update, except for four articles [34,51,62,66]. For these articles, the error elimination was performed by dismissing the updates that were detected to be acquired under NLOS.

### 3.3. Accuracy and Stability of Position Estimates

The position estimation errors are mostly reported as either mean (absolute) error MAE or RMSE, sometimes along with additional information like minimum error, maximum error, and error standard deviations. Some articles only provided error graphs (showing RMSE, MAE, or a cumulative error distribution function graph). Two articles reported the errors in MSE, which were converted to RMSE before listing them in Table 2. Among the articles reviewed, the smallest position estimation error based on MAE error was 0.04 m [38] and 0.076 m [33], respectively, while for RMSE the lowest reported position estimation error was 0.048 m [40], 0.066 m [7] and 0.068 m [39], respectively. Apart from this, the majority of reported errors were in the range between 0.1 m to 0.8 m, while four articles reported errors above 1 m of up to almost 2.5 m.

Most of the position estimation errors for the KF based-method were within 40 cm (nine out of seventeen articles) except for six articles [37,43,52,53,63,64] that had errors between 40 cm and 75 cm, and one article with errors as high as 2 m [51]. For the PF-based approach, only one article had an error below 15 cm which is 0.12/0.16 m [50]. For all the other PF-based articles, the errors were larger than 50 cm. The FIR-based articles had errors above 0.20 m and up to 0.78 m. For the graph optimization and combined KF/PF methods, the accuracies were above 0.4 m and above 0.5 m, respectively. Very few articles reported error standard deviation (SD). An overview of the position estimation accuracy along with the experiment details for all the reviewed articles is provided in Table 2. The reader may compare RMSE and MAE values under the assumption of a normally distributed error using their statistical relationship as explained in the Section 2 (RMSE = ~1.5 times MAE).

## 4. Discussion

### 4.1. General

Examining the number of records published over time, it appears that since 2014, there has been an upward trend in the number of publications fulfilling the search criteria for this study until the year 2020, with the years 2021 and 2022 being a major exception, and this result is possibly pandemic-related. However, there were still conference publications in these two years indicating that further research into this topic is happening that could still result in more publications in the near future.

This review’s main goal is to identify the possibilities and limitations of methods integrating UWB and MIMU sensor systems to provide accurate position estimates. To achieve this, four objectives were formulated in the Introduction. They were satisfied as follows: A summary of the current state-of-the-art UWB/MIMU integrated sensing for position estimation is provided along with a detailed description in the Section 3 of this paper, addressing objectives 1 and 2 (Section 3.2). Also, the achieved accuracies and stabilities reported in the reviewed articles were extracted and summarized addressing objective 3 (Section 3.3). This Discussion addresses the strengths and limitations of these methods in the context of the application of 3D analysis of human movement, addressing objective 4.

### 4.2. Position Estimation Accuracy and Stability

Among the reviewed articles, 20 articles (54%) have validated their position estimation accuracy only in clear LOS situations, while 12 articles (32%) validated their position estimation accuracy in a combination of LOS/NLOS situations. Only five articles (14%) validated their position estimation accuracies in both LOS and NLOS situations separately. In LOS situations only approximately seven articles (19%) of the total reviewed articles report errors of approximately 10 cm or less than 10 cm (Table 2). This is also only 26% of all the articles validated in the LOS situation. The highest accuracy results reported were an average position estimation error of 0.04 m in 2D position estimation [38] and an error of 0.048 m in 3D position estimation [40]. In addition to this, only two articles [7,39] reported 3D position estimate errors close to 0.05 m and less than 0.07 m, respectively. These four articles with the highest accuracies were all published between the years 2015 and the end of 2017. Apart from these, only three articles reported errors of approximately 0.1 m or less than 0.1 m, of which one was for 3D position estimate [41] while the other two were for 2D position estimates [33,66]. All other articles validated in LOS had errors higher than 0.13 m.

Amidst the seven articles reporting high accuracy, four articles were from the same author or research group (Zihajehzadeh et al.) who used information from an additional biomechanical model in their data fusion for the MIMUs alone, which could have helped in achieving better accuracy. The position error of article [40], with the lowest RMSE of 0.048 m, as well as the articles [49,62], was based on the validations in a slow activity with a very short measurement duration (i.e., 24 s and 10 s, respectively). How this method performs in longer recordings of more dynamic movements is not reported. Another author, Yoon et al., expressed doubts about the stability of these methods over longer periods [41].

The comparison of the results of the articles that validated the position estimation in NLOS situations is difficult since these errors very much depend on the type and dimensions of the obstruction, while these details are mostly unavailable in the articles reviewed. In general NLOS conditions, there is always an increased ranging estimate error that deteriorates the subsequent position estimation. None of the articles in NLOS had errors less than 10 cm. The highest accuracy reported in their specific NLOS situation was 0.128 m [66], 0.157 m [59], and 0.12/0.16 m (two different paths) [50], while all the other articles reported errors higher than 0.20 m for their own specific NLOS situations. Also, for article [66], the test duration was much shorter (approximately 10 s), which generate doubts about performance over a longer duration. Overall, the reported accuracies of the NLOS position estimate were widely varying, which is expected due to the varying NLOS conditions. Very little information was gathered from the reviewed studies on specific effects of NLOS situations as all obstructions were either environmental objects like pillars etc., or bodies of accidental passers-by in a corridor experiment. Only one study tried to calibrate for NLOS errors [50] based on the assumption that there is a fixed relationship between pose and error. This suggests a model for calibrating the structural component of the NLOS-related error.

As structural error components could possibly be mitigated by some sort of calibration method it is important to distinguish between random and structural components in the estimation errors. None of the other articles indicate the structural or random components for the reported errors. All authors reported accuracies in terms of estimation errors either expressed in RMSE or MAE. Only eight articles reported the estimation error standard deviation, representing the random component in the estimation error [32,39,50,53,54,58,63,67]. Still, the value of the structural component (bias) in the estimation error is not clear in any of these articles, as the average of the position errors was not explicitly mentioned. Also, it cannot be derived easily from reported RMSE and SD values which is the average error, as in all cases there seem to be both positive and negative error values.

For the successful application of this technology in the 3D analysis of human movement, the key criterion is the level of confidence that the clinicians can have in this system. This level of confidence or trustworthiness can be related to the validity and reliability of the sensor system [70]. The validity can be linked to the accuracy of the system. Meanwhile, the capability of the UWB/MIMU data fusion estimation methods to maintain the reported accuracy over longer recordings (consistency) can be related to the reliability of the system. From the observations of the reviewed articles, it can be concluded that the accuracy achieved so far is not close enough to the required targeted value of approximately 1 cm as stated in the Introduction (Section 1). Therefore, further improvements in accuracy are required for this integrated system to be useful for 3D analysis of human movement. The reliability parameter is supposed to be one of the major possible improvements of the integrated UWB/MIMU sensor system over MIMU-based methods. However, this accuracy over prolonged recordings (reliability) is not addressed or reported in any of the articles reviewed.

### 4.3. Effect on Position Estimation Accuracy Based on Sensor Configuration and Sensor Placement

Based on the sensor’s physical hardware integration, the situation with both the UWB and MIMU physically integrated into single hardware is called the ‘Integrated Hardware’ (IH) sensor, while when they were separate hardware systems it is called the ‘Non-integrated Hardware’ (NIH) sensor for this paper. The reviewed papers that use IH sensors in general report lower errors than 20 cm except for [61].Among the articles using NIH sensors, the articles that used physically tied NIH sensors (placed in the same location with the two sensor system synchronized), had more number of articles with position estimation errors lower than 20 cm (6 out of 15). Meanwhile, only two out of fifteen articles which used NIH sensors that are not physically tied to each other had errors lower than 20cm.

Although there are some exceptions, it seems that with IH sensors it is easier to achieve a higher accuracy, probably because their physical integration ties them together to a single location, which makes them experience the same kinematics and facilitates tightly synchronized data acquisition. Examining the accuracies as a function of the placement of the sensor on the body, the lowest errors were reported when using waist-mounted attachments for which all four articles reported errors of less than 11 cm. Followed by this, the two articles with shoulder placement reported errors of less than 16 cm. Among the more widely used locations (feet, shoulder bag, and hand) the feet had the highest accuracy with errors lower than 30 cm for most of the articles i.e., four out of seven articles.

Only one article mentioned the possibility of an effect on estimation accuracy of the sensor location on the body in its discussion [33]. No article reported on the effects on estimation accuracy caused by placement in different locations on the body. Outside the selected articles for this review, two articles by Otim et al. [71,72] studied the effect of the placement of a UWB sensor (without MIMUs) in multiple different locations on-body. These studies consist of UWB anchors placed around the test area (13 m × 6 m) and the UWB tags on different locations on the body. The distances measured are between the anchor and each tag on the body, while the positions of each tag on the body were estimated based on trilateration. In these two articles, they have studied the accuracies of the ranging and position for the following on-body locations namely forehead, hand, ankle, wrist, thigh, arm, and chest. From this study, the forehead is identified to be the location with the highest accuracy with average position errors of approximately 0.2 m and the chest is the location with worst accuracy with average position errors of approximately 2.46 m. The other locations between the forehead and chest in the descending order of accuracy were hands, ankle, wrist, thigh, and arm.

Direct comparison between these studies on locations [71,72] and the reviewed articles is not possible as the reviewed articles are the results of data fusion between the UWB and MIMU while the study of locations was performed only using the UWB sensors. However, if compared against the reviewed articles, assuming that the errors of UWB stay even after data fusion, the feet-mounted sensors had errors closer to the ankle-mounted situation and also for the hand-mounted situation it seems to be close enough. However, for the chest, in contrast to the findings of [71,72], the accuracy in reviewed articles was much lower and in the range between 0.23 m to 0.55 m.

### 4.4. Effect on Position Estimation Accuracy Based on the Data Fusion Algorithm/Methods

Out of the two data fusion approaches reported, the loosely coupled data fusion approach is claimed to be easier in implementation with less required computation time [37,38], and is used by approximately 49% of the articles reviewed and approximately 51% used the tightly coupled method. Loosely coupled approaches are stated to be susceptible to errors due to loss of information during the estimation of position from the measured UWB distances. The data fusion algorithm then has only these position estimates available in the update and possibly misses out on details that were present in the underlying UWB-based distance estimates. The tightly coupled approach is claimed to be beneficial over the loosely coupled approach since they utilize unprocessed distance estimates from the UWB for the data fusion algorithm [40]. However, of the seven articles reporting the highest accuracy, all except [40] used the loosely coupled approach. Also, analyzing the entire set of articles, similar accuracies are reported for both approaches. So, no evidence was found in the papers reviewed for the claims of possible higher achievable accuracy when using tightly coupled approaches. *This suggests that, based on currently published results, there indeed is no performance advantage of the tightly coupled approach, and therefore the loosely coupled approach seems preferable as it has the advantage of easier implementation and a lower computational cost.*

All seven articles with the highest reported accuracy used the KF-based method except [40], which used the MAP estimation algorithm (also the only article to use that method in very limited conditions). Other methods performed less well than the best six KF-based methods, which also were applied in more than 50% of the articles, *so based on this review KF seems the best candidate for achieving the high accuracy required for the analysis of human movement*.

All three articles [50,59,66] that reported the highest accuracies in NLOS conditions have used some form of explicit NLOS mitigation method in their algorithm. For the NLOS detection algorithm, both the methods based on communication channel characteristics and based on the ranging estimation inconsistencies are seen to be equally efficient in recognizing or identifying the NLOS situations. However, for the NLOS error-elimination methods, it is difficult to identify which error-elimination method is the best between the covariance-adjustment method and the method dismissing the NLOS updates. This is mainly attributed to the inconsistency in the accuracies reported for these methods, that is, both methods performed better in a few articles while having a lower accuracy in others. *Among the two methods mitigating the NLOS error, the covariance adaptation method seems to be a better strategy since it does not discard all the updates under NLOS conditions*.

### 4.5. General Recommendations

For integrated UWB/MIMU position estimation to be valuable for 3D analysis of human movement, achieving a high enough structural accuracy is the most important prerequisite. However, none of the studies reviewed reported an accuracy below, or close to, the targeted value of approximately 1 cm. Also, none of the studies explicitly report the actual magnitude of structural and random components in the errors. This is important as for both types of error components possible opportunities for improvement are very different in nature (e.g., structural components might be improved upon by improved calibration procedures and random components might be improved upon by increased redundancy in the number of ranging paths). Also, very little is reported about the stability of performance over longer recordings, which is important to understand their possible scope of application, especially as the main source of error in MIMU-only applications lies in time-variant integration drift errors, of which the magnitude is even depending on speed and type of movement performed. Therefore, future studies should separately examine and report structural and random errors, both as a function of recording duration and studied in all relevant movement scenarios. Also, none of the papers reported on the stability of the ranging accuracy over longer recordings and this should be further investigated.

The accuracy of the data fusion benefits from improved accuracy of UWB ranging, as the UWB-based distance estimates (or the derived position estimates) are serving as absolute time-invariant updates for the UWB/MIMU data fusion. Therefore, any further improvement of the UWB ranging estimates themselves will be beneficial for any future UWB/MIMU data fusion application. Most studies reviewed used the same UWB sensors from the same manufacturer ‘as is’. There was no mention of developing or performing custom calibration procedures to optimize the ranging performance of the UWB sensors used. As no, or very little, attention to these details is reported, it is not clear whether the optimal ranging performance is already achieved in any of the methods presented. This suggests that possibly UWB ranging performance improvement can be achieved by further investigation and optimizing the ranging estimate methods themselves including their calibrations methods. As NLOS situations would typically occur frequently in any 3D analysis of human movement application also studying their effects on ranging accuracy and ways of mitigation seems relevant.

## 5. Conclusions

This review provides a comprehensive analysis of the methods combining data from UWB and MIMU sensors mounted on a human subject for estimation of position. None of the articles reviewed reported an accuracy close to the desired 1 cm, which was stated to be required for successful application in the 3D analysis of human movement. The highest accuracies achieved in the LOS situation were reported in two articles to have an MAE of 0.04 m and an RMSE of 0.048 m, respectively, and both were achieved in rather limited conditions. All other articles reviewed reported substantially larger errors. The papers reviewed provided very little information on how large the contributions of structural and random components are to the estimation errors. This severely limits the possibilities of identifying possible opportunities for achieving the accuracies required for applications in the 3D analysis of human movement. For the different NLOS situations, the lowest errors reported were found to be approximately 0.12 m for both MAE and RMSE. NLOS conditions were clearly influencing the UWB ranging estimation performance. Still, reviewed articles revealed very little information on the nature and predictability of the extra errors of NLOS situations, which are especially relevant for application in the analysis of human movement. The effect of the mere presence of the human body on the accuracy of the position estimates is not reported or addressed, though some studies suggested there is a possible effect. This indicates a need for addressing the effect of these error sources in future research. Overall, this technical review intends to be a comprehensive resource offering insights into the current advancements and prospects of integrating UWB and MIMU sensors for accurate position estimation, especially for application in the field of 3D analysis of human movement.

## Figures and Tables

**Table 1:** Summary of information extracted from each reviewed article.

Ref. No.	Year	UWB Sensor	MIMU Sensor	UWB Config.	Update Rate [sps]		UWB Location	MIMU Location	UWB/MIMU Integration Method	UWB Update Param.	Sensor Attachment
					MIMU	UWB					
[37]	2012	-	-	4 Anchor and 1 Tag	200	3 to 4	Held in hand	Attached to the subject’s ankles	Loosely coupled EKF	Position	Not rigidly attached
[38]	2015	Ubisense Series 7000	Xsens MTx	4 Anchor and 1 Tag	100	10	Attached to the subject’s waist	Attached to the subject’s waist	Two-step cascaded KF	Position	Rigidly attached
[39]	2015	Ubisense Series 7000	Xsens MVN suit	4 Anchor and 1 Tag	100	16	Attached to the subject’s waist	One unit on the waist and six units for the right and left thighs, shanks, and feet.	Loosely coupled two-step Cascaded KF	Position	Not rigidly attached
[40]	2015	NA	Xsens	10 Anchor and 1 Tag	120	10	Attached to the subject’s foot	Attached to the subject’s foot	Maximum a Posteriori (MAP) estimation algorithm	Distance	Rigidly attached
[7]	2017	Unisense series 7000	Xsens MVN suit	4 Anchor and 3 Tag	100	10	Attached to the subject’s waist and both foot	One unit on the waist and six on the right and left thighs, shanks, and feet.	Multiple layered KF	Position	Rigidly attached
[41]	2017	Unisense series 7000	Xsens MTx	4 Anchor and 1 Tag	100	9.25	Attached to the subject’s waist	One unit on the waist, and six on the right and left thigh, shanks, and feet	Cascaded KF	Position	Rigidly attached
[42]	2017	-	-	3 Anchor and 1 tag	-	-	Attached to the subject’s shoulder	Attached to both of the subject’s feet	Unscented Kalman Filter (UKF)	Distance	Not rigidly attached
[32]	2017	DW1000	-	4 Anchor and 1 Tag	128	2	Attached to the helmet worn on head	Attached to the subject’s foot	PF	Position	Not rigidly attached
[43]	2018	BeSpoon	MATE9 (Huawei smartphone)	4 Anchor and 1 Tag	-	-	Held in hand	Held in hand	EKF	Distance	Rigidly attached
[31]	2018	DWM1000	X-IMU, UK MPU9250	4 Anchor and 1 Tag	128	3	Attached to the helmet worn on head	Attached to the subject’s foot	Method1: PF	Position	Not rigidly attached
									Method2: a combination of PF & EKF filter	Position	
[44]	2018	DWM1000	X-IMU, UK MPU9250	4 Anchor and 1 Tag	128	2	Attached to the helmet worn on head	Attached to the subject’s foot	Graph optimization	Position	Not rigidly attached
[45]	2018	-	-	4 Anchor and 1 Tag	-	-	Attached to a backpack setup carried by the subject	Attached to the subject’s foot	Federated Extended Finite Impulse Response (EFIR) filter	Distance	Not rigidly attached
[46]	2018	DW1000	Acc:ADXL203;Gyr:ADXRS620;Mag:HMC5983	5 Anchor and 1 Tag	-	-	Attached to a backpack setup carried by the subject	Attached to the subject’s foot	UFIR filter bank	Position	Not rigidly attached
[47]	2019	EVB1000/DW1000	Iphone IMU	1 Anchor and 1 Tag	50	10	Held in hand	Held in hand	PF	Distance	Rigidly attached
[48]	2019	-	-	4 Anchor and 1 Tag	100	-	Attached to a backpack setup carried by the subject	Attached to the subject’s foot	Predictive Adaptive Kalman Filter (PAKF)	Distance	Not rigidly attached
[49]	2019	-	Acc:ADXL203;Gyr:ADXRS620;Mag:HMC5983	5 Anchor and 1 Tag	-	-	Attached to a backpack setup carried by the subject	Attached to the subject’s foot	Predictive UFIR filter	Position	Not rigidly attached
[50]	2019	DW1000	iPhone IMU	3 Anchor and 1 Tag	50		Held in hand	Held in hand	PF	Distance	Rigidly attached
[51]	2019	DW1000	LSM330DLC;Mag:HMC5883L	3 Anchor and 1 Tag	100	1 to 2	-	-	EKF	Position	-
[52]	2019	-	JY901B	4 Anchor and 1 Tag	-	1	Held in hand	-	EKF	Position	-
[53]	2019	-	MPU9150	7 Anchor and 1 Tag	100	1	Attached to the helmet worn on head	Attached to the subject’s foot	Iterative EKF	Distance	Not rigidly attached
[33]	2020	DWM1000	MPU9250	3 Anchor and 1 Tag	-	-	Attached to the subject’s foot	Attached to the subject’s foot	KF	position	Rigidly and integrated hardware
[54]	2020	UWB Mini4Plus	JY61 attitude sensor	4 Anchor and 1 Tag	-	-	Attached to the subject’s foot	Attached to the subject’s foot	EKF	position	Rigidly attached
[55]	2020	-	-	4 Anchor and 1 Tag	100	-	Attached to a backpack setup carried by the subject	Attached to the subject’s foot	Decision Tree-EFIR filter	Distance	Not rigidly attached
[56]	2020	-	-	4 Anchor and 1 Tag	-	-	Attached to a backpack setup carried by the subject	Attached to the subject’s foot	Least Square-Support Vector Machine (LS- SVM) assisted UFIR filter	Position	Not rigidly attached
[57]	2020	EVB1000/DW1000	iPhone 7 IMU	3 Anchor and 1 Tag	50	3.57	Held in hand	Held in hand	PF	Distance	Rigidly attached
[58]	2020	EVB1000/DW1000	iPhone 7 IMU	2 Anchor and 1 Tag	50	3.57	Held in hand	Held in hand	PF	Distance	Rigidly attached
[57]	2020	EVB1000/DW1000	iPhone 7 IMU	2 Anchor and 1 Tag	50	3.57	Held in hand	Held in hand	PF	Distance	Rigidly attached
[35]	2020	DWM1000/MAX2000	LIS3DH	3 Anchor and 1 Tag	-	-	-	-	EKF	Distance	-
				1 Anchor and 1 tag					UKF		-
[34]	2020	PlusOn410	Starneto, China	5 Anchor and 1 Tag	100	10	Attached to the subject’s foot	Attached to the subject’s foot	KF	Position	Rigidly attached
[59]	2020	DWM1000	Acc. & Gyr.:ICM20602;Mag: IST8310	3 Anchor and 1 Tag	200	20	Attached to the subject’s shoulder	Attached to the subject’s shoulder	KF	Distance	Rigidly and integrated hardware
[60]	2021	DW1000	Acc:ADXL203;Gyr:ADXRS620;Mag:HMC5983	5 Anchor and 1 Tag	30	3	Attached to a backpack setup carried by the subject	Attached to the subject’s foot	EFIR filter	Distance	Not rigidly attached
[61]	2021	MAX2000/DW1000	MPU6050	4 Anchor and 1 Tag	-	-	Attached to the subject’s foot	Attached to the subject’s foot	tightly-coupled EKF	Distance	Rigidly and integrated hardware
[62]	2021	DW1000	EBIMU-9DOF	4 Anchor and 1 Tag	50	16	Held in hand at a constant location close to the chest	Held in hand at a constant location close to the chest	KF	Position	Rigidly attached
[63]	2021	-	MPU9250	5 Anchor and 1 Tag	100	2	Attached to the subject’s foot	Attached to the subject’s foot	EKF	Distance	Rigidly attached
[64]	2021	-	Acc:ADXL203;Gyr:ADXRS620;Mag:HMC5983	4 Anchor and 1 Tag	-	-	Attached to a backpack setup carried by the subject	Attached to the subject’s foot	Distributed KF	Distance	Not rigidly attached
[65]	2022	DWM1001C	MPU6050 InvenSense, San Jose, CA, USA	4 Anchor and 1 Tag	100	10	Attached to the subject’s foot	Attached to the subject’s foot	loosely coupled EKF	Position	Rigidly attached
[66]	2023	DW1000	-	4 Anchor and 1 Tag	-	-	Body worn; On-body location not mentioned	Body worn; On-body location not mentioned	KF	Position	-

- data not specified in the reviewed articles.

**Table 2:** Accuracy of position estimates and stability parameters.

Ref. No.	2D/3D	LOS/NLOS	Exp. Activities	Integrated Accuracy [m]				Errors Around Each Axis [m]	Test Time [s]
				RMSE	MAE	Min Error	Max Error		
[37]	2D	Combined LOS and NLOS	Walking	-	0.4	-	-	-	~240
[38]	2D	LOS	Jumping task	-	<0.04	-	-	<0.04 in x and y axis	90
[39]	3D	LOS	Walking and Jumping	Walking: 0.068 * Jumping: 0.073 *	-	-	-	Walking: X-0.039 ± 0.016; Y-0.036 ± 0.015; Z-0.043 ± 0.017 Jumping: X-0.042 ± 0.019; Y-0.036 ± 0.015; Z-0.049 ± 0.023	~120
[40]	3D	LOS	Walking	0.048 *	-	-	-	X-0.03; Y-0.03; Z-0.023	24
[7]	3D	LOS	Walking and Jumping	Waist: 0.075 * Feet: 0.067 *	-	-	-	Waist: X-0.043; Y-0.048; Z-0.038; Feet: X-0.039; Y-0.041; Z-0.035	100
[41]	3D	LOS	Walking and Dynamic (combining Running and Jumping)	Overall: 0.108 Walking: 0.092 Dynamic: 0.129	-	-	-	Overall: X-0.074; Y-0.072; Z-0.030; Walking: X-0.063; Y-0.062; Z-0.026; Dynamic: X-0.086; Y-0.086; Z-0.04	75
[42]	2D	LOS	Walking at different speeds from 1–3 m/s	-	Overall: 0.15 Speed 1 m/s: 0.129 Speed 2 m/s: 0.155 Speed 3 m/s: 0.195	0.05	0.35	-	30
[32]	2D	Separate LOS and NLOS	Walking	-	LOS: 0.708 ± 0.660 NLOS: 0.726 ± 0.661	-	-		~24 to 58
[43]	2D	Combined LOS and NLOS	Walking along two different routes	Route 1 (Less NLOS): 0.35 Route 2 (More NLOS): 0.45	-	-	-	-	-
[31]	2D	Separate LOS and NLOS	Walking along two different routes	-	LOS Route 1: 0.637 Route 2: 0.531 NLOS Route 1: 0.735 Route 2: 0.571	LOS Route 1: 0.001 Route 2: 0.001 NLOS Route 1: 0.003 Route 2: 0.007	LOS Route 1: 2.087 Route 2: 1.462 NLOS Route 1: 2.896 Route 2: 1.816	-	~390 to 420
				-	LOS Route 1: 0.685 Route 2: 0.505 NLOS Route 1: 0.624 Route 2: 0.527	LOS Route 1: 0.003 Route 2: 0.009 NLOS Route 1: 0.003 Route 2: 0.008	LOS Route 1: 2.576 Route 2: 1.356 NLOS Route 1: 2.576 Route 2: 1.524	-	
[44]	2D	Combined LOS and NLOS	Walking along three different routes	-	Route 1: 0.413 Route 2: 0.369 Route 3: 0.372	-	-	-	~100 to 180
[45]	2D	LOS	Walking	0.576 *	-	-	-	X-0.36; Y-0.45	45
[46]	2D	LOS	Walking	0.297 *		-	-	X-0.2; Y-0.22	60
[47]	2D	LOS	Walking along two different routes	-	Route 1: 0.60 Route 2: 0.58	-	-	-	258.8 & 391.8
[48]	2D	LOS	Walking	0.299 *	-	-	-	X-0.173 **; Y-0.245 **	30
[49]	2D	LOS	Walking along three different routes	Route 1: 0.391 * Route 2: 0.353 * Route 3: 0.700 *	-	-	Route 1: X-1.03; Y-1.21 Route 2: X-0.44; Y-1.20 Route 3: X-2.28; Y-2.20	Route 1: X-0.25; Y-0.30 Route 2: X-0.15; Y-0.32 Route 3: X-0.50; Y-0.49	25
[50]	2D	Combined LOS and NLOS	Walking along two different routes	-	Route 1: 0.125 ± 0.059 Route 2: 0.164 ± 0.084	-	-	-	50 & 100
[51]	2D	Separate LOS and NLOS	Walking	-	LOS: less than 1.5 m for 99th percentile; NLOS: Less than 2 m for the 99th percentile	-	-	-	-
[52]	2D	Combined LOS and NLOS	NA	-	~0.4	0.05	-	-	-
[53]	2D	Combined LOS and NLOS	Walking along two different routes	-	Route 1: 7-anchor: 0.58 ± 0.22 3-Anchor: 0.62 ± 0.33 2-anchor: 0.63 ± 0.34 Route 2: 7-anchor: 0.59 ± 0.27 3-Anchor: 0.66 ± 0.32 2-anchor: 0.96 ± 0.47	-	NA		180 & 585
[33]	2D	LOS	Walking	-	0.076	-	-	X-0.051; Y-0.055	~20 to 25
[54]	2D	Separate LOS and NLOS	Walking	-	LOS: 0.24 ± 0.26 NLOS: 0.35 ± 0.35	-	LOS: 1.52 NLOS: 1.02	LOS: X-0.30; Y-0.18 NLOS: X-0.43; Y-0.23	~20 to 40
[55]	2D	LOS	Walking	0.788 *	-	-	-	X-0.364; Y-0.699	~20 to 25
[56]	2D	LOS	Walking	0.264 *	-	-	-	X-0.173 **; Y-0.200 **	~270
[57]	2D	Combined LOS and NLOS	Walking along two different routes	-	Route 1: 0.87 ± 0.52 Route 2: 0.81 ± 0.39	-	-	-	323 & 447
[58]	2D	Combined LOS and NLOS	Walking	-	2.09 ± 1.33	-	-	-	~479
[57]	2D	LOS	Walking	-	2.48	-	-	-	334.1 & 329.9
[35]	2D	LOS	NA	< 0.2	-	-	-	-	NA
				<0.16	-	-	-	-	
[34]	2D	LOS	Walking	0.132	-	-	-	-	~550
[59]	2D	Combined LOS and NLOS	Walking	-	0.157	-	0.601	-	~50
[60]	2D	LOS	Walking	0.305 *	-	-	-	X-0.20; Y-0.23	90
[61]	3D	LOS	Walking	-	50% of the time below 0.39	-	-	-	325
[62]	2D	Combined LOS and NLOS	Walking along six different routes	Less NLOS Route 1: 0.234; Route 2: 0.39; Route 3: 0.556 More NLOS Route 4: 0.314; Route 5: 0.492; Route 6: 0.473 Average: 0.4266	-	-	-	-	~15 to 30
[63]	2D	Combined LOS and NLOS	Walking along two different routes	-	Route 1: 0.48 ± 0.37 Route 2: 0.62 Reduced no of Anchors: 4Anc: 0.36 ± 0.24; 3Anc: 0.51 ± 0.24 (Best combi)	-	-	-	250
[64]	2D	LOS	Walking along two different routes	Route 1: 0.61 Route 2: 0.53	-	-	-	-	460
[65]	2D	Combined LOS and NLOS	Walking along two different routes	-	Route 1: 0.24 Route 2: 0.29	-	Route 1: 0.47 Route 2: 0.66	Route 1: X-0.18; Y-0.15 Route 2: X-0.18; Y-0.24	~10 to 20
[66]	2D	Separate LOS and NLOS	Walking along two different routes	NLOS: 0.128 LOS: 0.099	-	-	-	-	~5 to 10

* Estimated position RMSE from the corresponding RMSE errors around each axis; ** RMSE computed from provided MAE values.

## Data Availability

Not applicable.

## Abbreviations

EFIR	Extended Finite Impulse Response
EKF	Extended Kalman Filter
FIR	Finite Impulse Response
IH	Integrated Hardware
KF	Kalman Filter
LOS	Line of Sight
MAE	Mean Absolute Error
MAP	Maximum a Posteriori
MIMU	Magnetic Inertial Measurement Unit
MSE	Mean Square Error
NIH	Non-integrated Hardware
NLOS	Non-Line-of-Sight
PDR	Pedestrian Dead Reckoning
PF	Particle Filter
RFID	Radio Frequency Identification
RMSE	Root Means Square Error
RSS	Received Signal Strength
ToA	Time of Arrival
TWR	Two-Way Ranging
UFIR	Unbiased Finite Impulse Response
UKF	Unscented Kalman Filter
UWB	Ultra-wideband
ZUPT	Zero Velocity Update algorithm
